# Clinical and Echographic Features of Morning Glory Disc Anomaly in Children: A Retrospective Study of 249 Chinese Patients

**DOI:** 10.3389/fmed.2021.800623

**Published:** 2022-01-24

**Authors:** Yihua Zou, Kaiqin She, Yiqian Hu, Jianing Ren, Ping Fei, Yu Xu, Jie Peng, Peiquan Zhao

**Affiliations:** ^1^Department of Ophthalmology, Xinhua Hospital Affiliated to Shanghai Jiao Tong University School of Medicine, Shanghai, China; ^2^Department of Ophthalmology, West China Hospital, Sichuan University, Chengdu, China

**Keywords:** morning glory disc anomaly, retinal detachment, visual acuity, retrospective study, ultrasound technique

## Abstract

**Purpose:**

To report the clinical and echographic features, the prevalence of retinal detachment (RD), and associated visual acuity in a cohort of pediatric patients with morning glory disc anomaly (MGDA).

**Methods:**

This was a retrospective review of 249 pediatric patients with MGDA (271 eyes) seen at the Dept. of Ophthalmology, Xinhua Hospital. Their medical records were reviewed for demographic data and ocular and systemic findings. The maximal depth and width of the cavity were measured using standardized echographic images. The ratios of cavitary depth to axial length, cavitary depth to maximal cavitary width, and the product of cavitary depth and width were calculated and used to indicate the relative size of the excavation. The clinical and echographic findings were correlated with visual acuity and the occurrence of RD of the patient.

**Results:**

The relative size of the excavation and the presence of RD were positively associated with increased risk of poor vision (*p* < 0.05). The presence of persistent fetal vasculature was not associated with the risk of RD and poor vision. The ratio of cavitary depth to axial length more than or equal to 0.25 conferred an increased risk of RD (OR, 2.101; 95% CI, 1.469–3.003).

**Conclusions:**

Clinical and echographic features of MGDA may be used in predicting the risk of RD. Measuring the relative size of excavation *via* echography may guide the follow-ups and assist in the early diagnosis of RD.

## Introduction

Morning glory disc anomaly is a rare congenital cavitary anomaly of the optic disc, characterized by an enlarged and excavated optic disc with juxtapapillary chorioretinal pigment disturbance, radial retinal blood vessels, and a central white glial tuft. It was first named by Kindler in 1970 because of its resemblance to the morning glory (1). Its prevalence is ~3.6 per 100,000 children (2). The visual acuity of the affected children is usually poor, <0.1 of the decimal visual acuity. The exact pathogenesis of MGDA remains unknown but may be related to poor development of the posterior sclera and lamina cribrosa (3).

Patients with MGDA often have other abnormalities. Ocular complications, such as persistent fetal vasculature (PFV), cataracts, microphthalmia, and retinal detachment (RD), are frequently found in MGDA eyes. Systemic disorders, such as Aicardi syndrome, basal encephalocele, persisting embryonal infundibular recess, and other cerebrovascular anomalies, have been reported together with MGDA (2, 4–7). Eyes with MGDA and PFV have been well-described by Fei et al. (8), which showed a high occurrence of ocular complications. However, studies on visual prognosis of MGDA eyes with variable clinical features are rare.

About one-third of MGDA eyes had RD at diagnosis (9), which can follow an unpredictable clinical course of spontaneous attachment and re-detachment (10). Surgeries aimed to reattach the retina is challenging. Sometimes, multiple surgeries were needed, especially in eyes with advanced RD (11). Despite anatomical success, visual prognosis is always worse than before. Finding relative risk factors of RD is quite needed for surgeons to guide the follow-ups in children and diagnose the occurrence of RD earlier.

In the current study, we evaluated the excavation in a cohort of 271 MGDA eyes based on ocular echography using a method proposed by Venincasa et al. for ocular coloboma (12). The association between clinical and echographic features of MGDA and the risk of RD and visual prognosis were then analyzed. To the best of our knowledge, this is the first study that used echography to evaluate the prognosis of MGDA.

## Materials and Methods

This retrospective study was approved by the Institutional Review Board of the Xinhua Hospital affiliated to Shanghai Jiao Tong University School of Medicine. The study was conducted in compliance with the Declaration of Helsinki. A total of 249 patients diagnosed with “morning glory syndrome” or “morning glory disc anomaly” between July 2010 and August 2021 in the Department of Ophthalmology of Xinhua Hospital were included. The patients with optic nerve coloboma were excluded. Patient charts were reviewed and the data collected included: demographics, major symptoms (strabismus, nystagmus, leukocoria, smaller eye size, decreased vision, abnormal fundus presentation, and others), ultrasound measurement of maximal cavitary depth and width, presence of classic PFV and their types (anterior, posterior, and combined), visual acuity, presence of RD, other ocular conditions (microphthalmia, glaucoma, and cataract), and non-ocular conditions (developmental delay, intellectual disability, cleft lip, and cranial abnormalities, including Moyamoya disease, cerebral artery stenosis, callosal agenesis, and so forth). Although all the patients were asked to have cranial magnetic resonance imaging (MRI) and MR angiography (MRA) or computed tomography (CT) examinations, only 75 such reports were available.

When applicable, Snellen fraction and non-numerical visual acuity values were converted to a numerical form (logarithm of the minimum angle of resolution, logMAR), permitting statistical analysis. As described by Moussa et al. (13), “counting fingers” (CF) was equivalent to logMAR 2.1; “hand motion” (HM) was equivalent to logMAR 2.4; “light perception” (LP) was equivalent to logMAR 2.7; and “no light perception” (NLP) was equivalent to logMAR 3.0.

**Figure 1:**
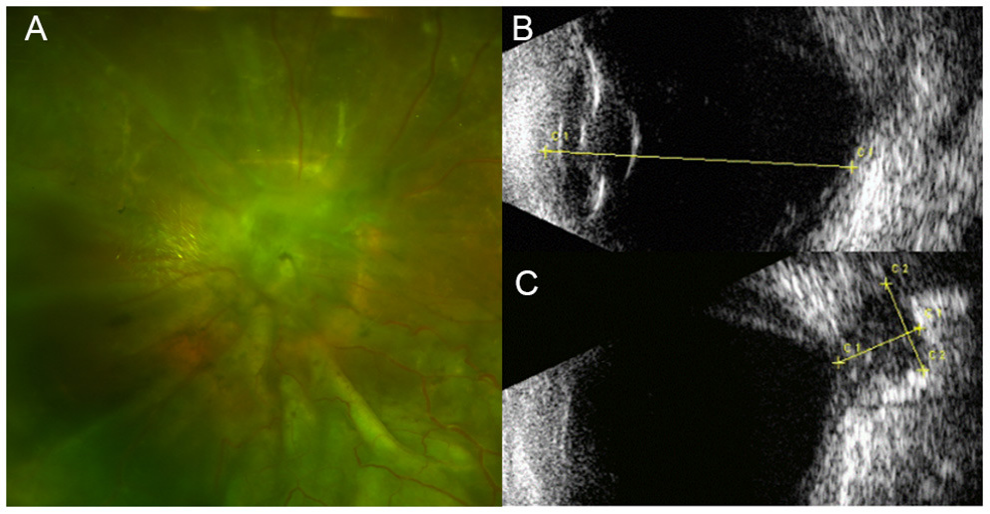
One sample of echographic measurement with digital images. **(A)** Fundus image of one eye of morning glory disc anomaly (MGDA) with retinal detachment (RD). **(B)** The axial length (AL) was measured from the anterior apex of cornea to the anterior of retina close to the margin of excavation. **(C)** The maximal cavitary depth (C1-C1) and the maximal cavitary width (C2-C2) were measured at the maximal cross section of the excavation.

**Figure 2:**
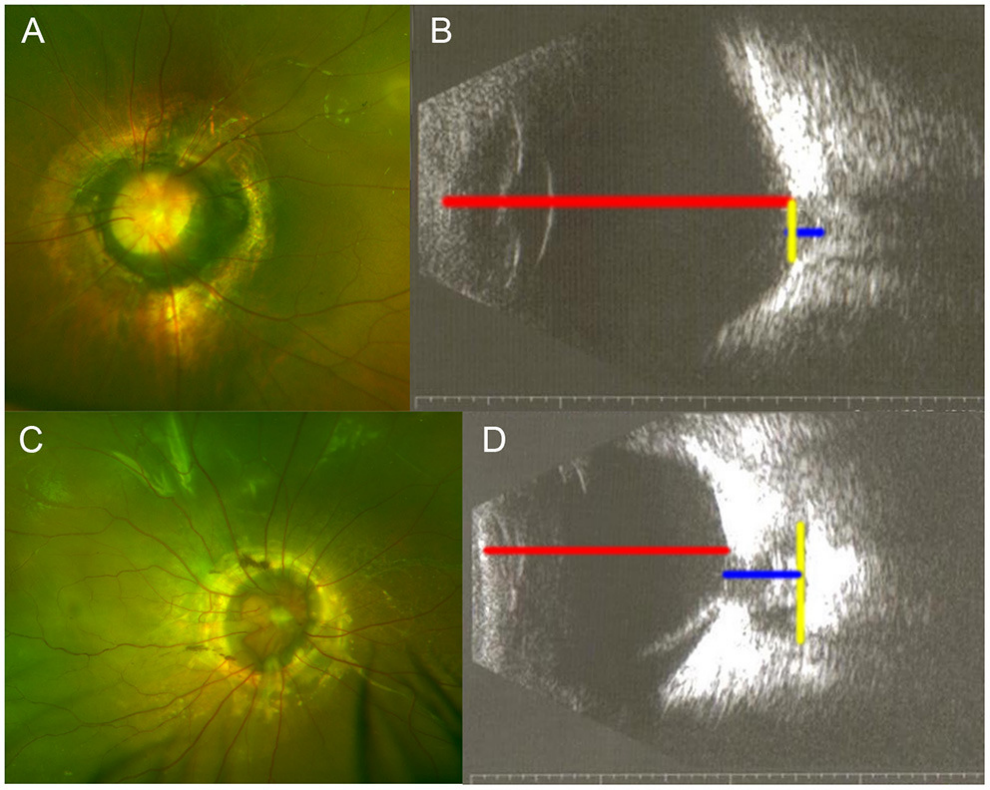
Samples of echographic measurement with printed images. **(A,B)** One eye of morning glory disc anomaly (MGDA) without retinal detachment (RD). **(C,D)** One eye of MGDA with RD. Red line, axial length; blue line, maximal cavitary depth; yellow line, maximal cavitary width.

The echographic parameters were measured as described by Venincasa et al. (12). In brief, the parameters of the eye were measured with a portable ultrasound probe (mode: B scan, Aviso or CineScan, Quantel Medical, Clermont-Ferrand, France). The axial length, the maximal depth, and the maximal width of each excavation were measured to the nearest millimeter with calipers from the digital or printed images (Figures 1, 2). The axial length was defined as the linear distance between the anterior apex of the cornea and the margin of the excavation. When measuring the axial length, the depth of the cavity was not included (Figures 1, 2). The maximal cavitary depth was defined as the measurement between the apex and the base of the excavation. Given the variable morphology of the excavation, the volume was not calculated. Instead, the relative size of the excavation was represented by the following parameters: the ratio of cavitary depth to axial length, the ratio of cavitary depth to maximal cavitary width, and product of cavitary depth and width. Microphthalmia was defined as an eye with axial length less than 2 SD below the mean for that age (axial length < 16 mm at birth, < 19 mm at 1 year) (14). All parameters of each excavation were performed by the same technician.

Statistical analyses were performed with SPSS 22.0 (SPSS, Inc., Chicago, IL, USA) statistical software. Student's *T*-test, Mann–Whitney *U*-test, and chi-square test were used to compare the numerical or categorical variables with the presence or lack of RD and visual acuity better or worse than 2/200 (logMAR 2.0). Cox regression was used in the analysis of risk factors for RD. A *P*-value of equal or less than 0.05 was considered as statistically significant.

## Results

A total of 271 MGDA eyes of 249 patients were included in this study. Bilateral MGDAs were present in 22/249 (8.8%) patients. The mean age at diagnosis was 30.3 ± 28.6 months (median = 22.1; range, 0.2–160.3 months); the mean age at last follow-up was 51.2 ± 33.2 months (median = 48; range, 2.3–182 months). The major symptoms at diagnosis included leukocoria (6%), esotropia (13%), exotropia (14%), smaller eye size (11%), decreased vision (15%), and nystagmus (6%). Patient demographics are shown in Table 1.

**Table 1:** Patient demographics.

No. patients (% female)	249 (50.6)
No. MGDA (% bilateral)	271 (8.8)
Symptoms at diagnosis	
Leukocoria	14 (6)
Esotropia	32 (13)
Exotropia	36 (14)
Smaller eye size	28 (11)
Decreased vision	38 (15)
Nystagmus	15 (6)
Av. age at diagnosis, mean m ± SD, median (range)	30.3 ± 28.6, 22.1 (0.2–160.3)
Av. age at follow-up, mean m ± SD, median (range)	51.2 ± 33.2, 48.0 (2.3–182.0)
Av. Time from diagnosis to follow-up, mean m ± SD, median (range)	20.9 ± 24.6, 11.2 (0–123.3)

**Figure 3:**
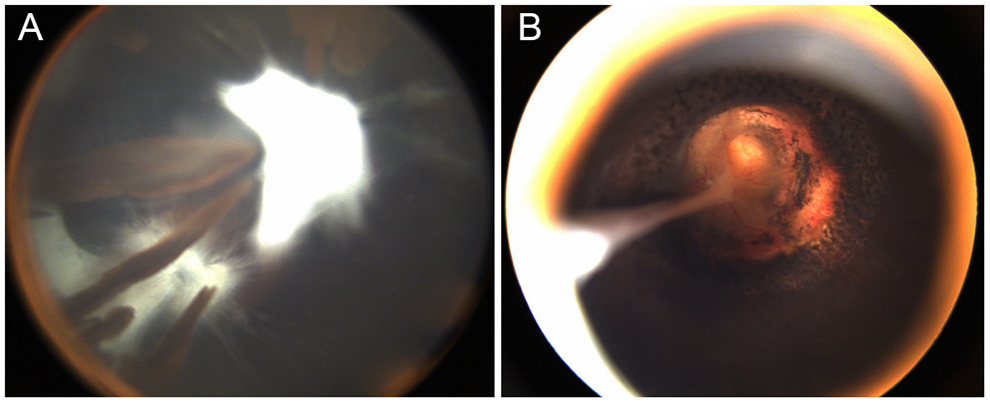
One patient with morning glory disc anomaly associated with combined persistent fetal vasculature (PFV). **(A)** The classic PFV with a prolonged ciliary process; **(B)** The MGDA excavated optic disc and residual cord rising from the disc to the posterior of the capsule after lensectomy and laser coagulation around the optic disc.

**Figure 4:**
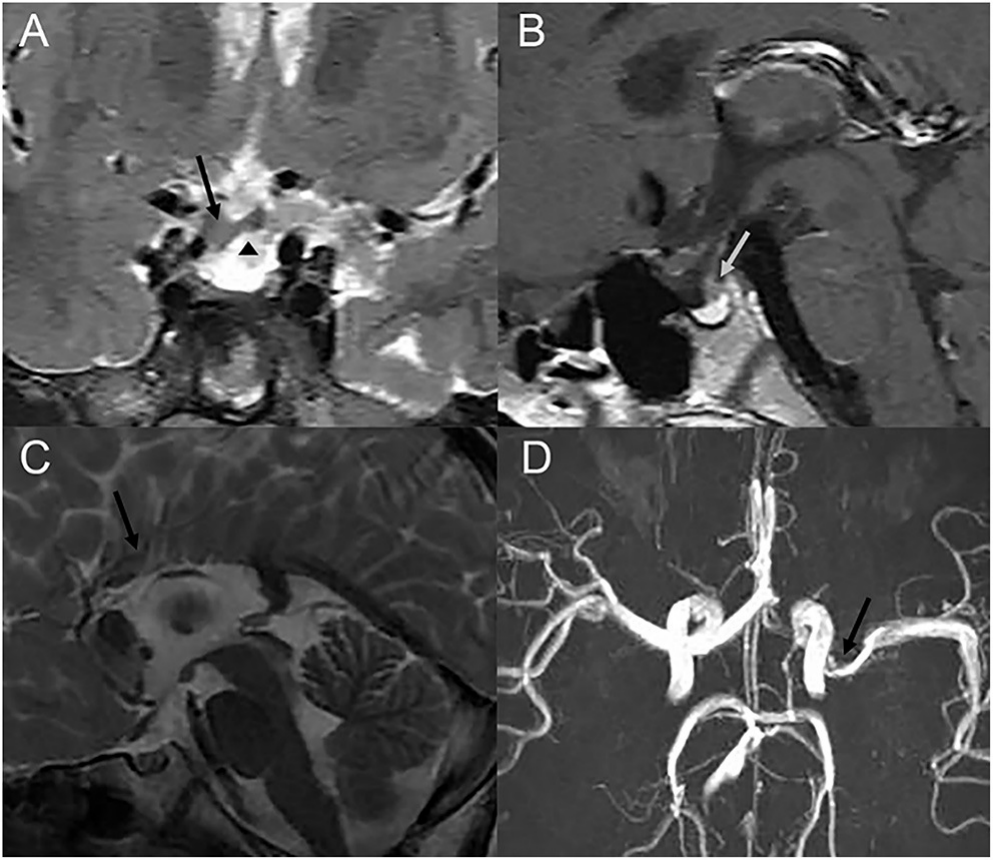
Cranial MRI or MRA images of three patients with cranial abnormalities. **(A,B)** Coronal T2-weighted and sagittal T1-weighted images of one patient, who showed growth hormone deficiency, showing a thickened optic chiasm (a black arrow), an ectopic pituitary (a triangle), and abnormal pituitary stalk (a grey arrow). **(C)** Sagittal T2-weighted images of one patient showing the corpus callosum body agenesis. **(D)** Cranial MRA showing the narrowing of the left M1 MCA segment (an arrow).

Other associated ocular and non-ocular abnormalities are presented in Table 2. Classic PFV (Figure 3) was found in 77 eyes (28% of total eyes), and microphthalmia was found in 42 eyes (15%). The most common secondary complication was cataract, which was found in 23 eyes (8%), followed by secondary glaucoma in three eyes (1%). Developmental delay was present in three patients and intellectual disability in one patient. Cranial MRI/MRA or CT examinations from 75 patients showed abnormalities in 8 patients, including Moyamoya disease in one patient, cerebral artery stenosis in three patients, callosal agenesis in one patient, arachnoid cyst in one patient, and pituitary dysplasia in two patients (Figure 4).

**Table 2:** Patient ocular and non-ocular anomalies.

	***n* (%)**
Persistent fetal vasculature (PFV), *n* = 271	
Anterior PFV	36 (13)
Combined PFV	41 (15)
Posterior PFV	0 (0)
Cataract, *n* = 271	23 (8)
Microphthalmia, *n* = 271	42 (15)
Glaucoma, *n* = 271	3 (1)
Abnormal development, *n* = 249	
Developmental delay	3 (1)
Intellectual disability	1 (0.4)
Cleft lip	1 (0.4)
Cranial anomalies, *n* = 75	
Moyamoya disease	1 (1)
Cerebral artery stenosis	3 (4)
Callosal agenesis	1 (1)
Arachnoid cyst	1 (1)
Pituitary dysplasia	2 (3)

Axial length, cavitary depth, and maximal cavitary width were measured based on digital or printed echographs (Figures 1, 2). The ratio of cavitary depth to axial length, cavitary depth to maximal cavitary width, and the product of cavitary depth and width were calculated and used as indicators of the relative size of the cavity. The average values of these parameters presented are listed in Table 3.

**Table 3:** Echographic characteristics of MGDA in this study (*n* = 271).

Av. axial length, mean mm ± SD, median (range)	20.25 ± 2.32, 20.4 (11.0–28.5)
Av. cavitary depth, mean mm ± SD, median (range)	4.79 ± 2.07, 4.7 (1.2–13)
Av. cavitary width, mean mm ± SD, median (range)	5.69 ± 2.2, 5.3 (1.4–16.6)
Av. cavitary depth/axial length, mean ± SD, median (range)	0.24 ± 0.11, 0.23 (0.05–0.68)
Av. cavitary depth/cavitary width, mean ± SD, median (range)	0.88 ± 0.31, 0.83 (0.30–3.47)
Av. product of cavitary depth and width, mean ± SD, median (range)	30.81 ± 25.25, 23.65 (1.82–156)

### Correlation Between Echographic Parameters and Poor Vision

Vision acuity was measured in 113 eyes (42%, Table 4). The rest of the eyes (158 eyes) had no data on vision acuity due to extremely young age of the affected patients (16.6 ± 15.9 months). Increased cavitary depth and the relative size of excavation, and the presence of RD were significantly associated with an increased risk of poor vision (all *p* < 0.05). Cavitary width, the product of cavitary depth and width, and the presence of PFV were not associated with the risk of poor vision. However, there was a trend toward increasing risk of poor vision with the presence of PFV (0.17 in eyes with VA of better than 2/200 vs. 0.25 in eyes with VA of worse than 2/200, *P* = 0.433).

**Table 4:** Best-corrected visual acuity (LogMAR) at follow-up.

	**Worse than 2/200, *n* = 95**	**Better than 2/200, *n* = 18**	***P*-value**
Cavitary depth, mean mm ± SD, median (range)	5.04 ± 1.99, 4.7 (1.9–8.6)	4.02 ± 2.75, 2.9 (1.3–10.3)	0.028
Cavitary width, mean mm ± SD, median (range)	5.63 ± 2.16, 5.3 (2.8–10.7)	5.73 ± 3.15, 5.0 (1.8–12.8)	0.369
Cavitary depth/axial length, mean ± SD, median (range)	0.25 ± 0.11, 0.22 (0.09–0.48)	0.18 ± 0.13, 0.13 (0.06–0.52)	0.008
Cavitary depth/cavitary width, mean ± SD, median (range)	0.96 ± 0.36, 0.93 (0.30–1.63)	0.68 ± 0.21, 0.66 (0.35–1.13)	0.006
Product of cavitary depth and width, mean ± SD, median (range)	29.75 ± 17.48, 26.39 (5.32–58.48)	30.30 ± 35.19, 13.88 (2.40–114.15)	0.070
Retinal detachment present, *n* (%)	65 (68)	5 (28)	0.001
PFV present, *n* (%)	24 (25)	3 (17)	0.433

### Correlation Between Echographic Parameters and RD

RD was present in 129/271 eyes (48%, Table 5). Increased cavitary depth, cavitary width, and relative size of excavation were all significantly associated with an increased risk of RD (all *p* < 0.05), and decreased axial length was significantly associated with an increased risk of RD (*p* < 0.05). The presence of PFV was not associated with RD (*P* = 0.656). Complete data on RD are shown in Table 5.

**Table 5:** Demographic, echographic, and structural characteristics of eyes with and without retinal detachment.

	**Yes, *n* = 129**	**No, *n* = 142**	***P*-value**
Age at RD, mean m ± SD, median (range)	59.64 ± 32.61, 58.6 (4.5–154.6)		
Cavitary depth, mean ± SD, median (range)	5.45 ± 1.92, 5.3 (1.2–13.0)	4.24 ± 2.07, 3.7 (1.2–11.8)	0.000
Cavitary width, mean ± SD, median (range)	6.20 ± 2.32, 5.9 (1.4–15.4)	5.22 ± 2.41, 4.6 (1.7–16.6)	0.000
Cavitary depth/axial length, mean ± SD, median (range)	0.28 ± 0.11, 0.27 (0.06–0.68)	0.21 ± 0.11, 0.18 (0.05–0.56)	0.000
Cavitary depth/cavitary width, mean ± SD, median (range)	0.92 ± 0.26, 0.86 (0.42–1.63)	0.85 ± 0.36, 0.79 (0.30–3.47)	0.009
Product of cavitary depth and width, mean ± SD, median (range)	36.79 ± 25.30, 32.76 (1.82–156.00)	25.53 ± 24.04, 16.65 (2.40–139.24)	0.000
Axial length, mean ± SD, median (range)	19.53 ± 2.34, 19.6 (11.0–25.8)	20.84 ± 2.02, 21.0 (15.5–26.5)	0.000
PFV present, *n* (%)	35 (27)	42 (30)	0.656

**Figure 5:**
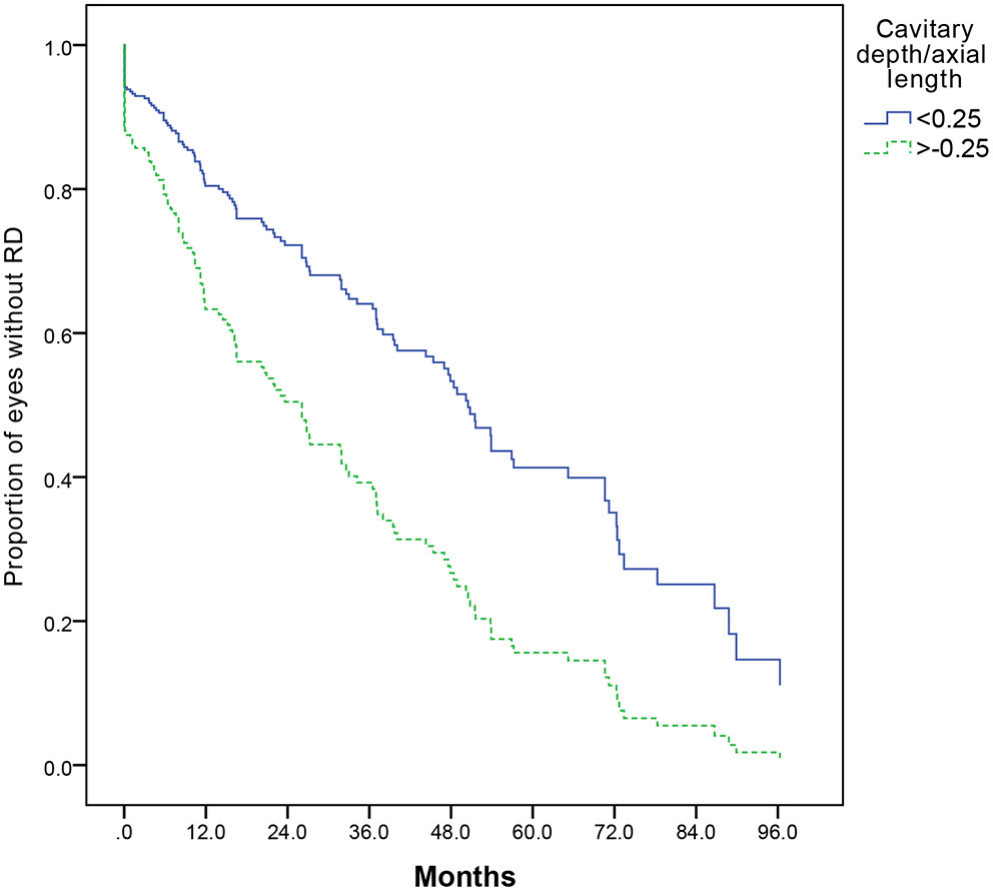
A Kaplan–Meier plot comparing risk of retinal detachment with cavitary depth/axial length more than or equal to, or less than 0.25 (*P* = 0.000). A Cox regression analysis, including cavitary depth, cavitary width, relative size of excavation (cavitary depth/axial length, cavitary depth/cavitary width, and product of cavitary depth and width), and the presence of PFV, is performed, and cavitary depth/axial length is the only statistically significant risk factor (*P* = 0.000).

Cox regression analysis was used to evaluate the risk factors of RD. Increased cavitary depth to the axial length ratio was associated with an increased risk of RD (*P* = 0.000). Further analysis showed that the cavitary depth to the axial length ratio of more than or equal to 0.25 was associated with an increased risk of RD compared to those with the ratio of less than 0.25 (OR, 2.101; 95% CI, 1.469–3.003) (Figure 5).

## Discussion

The visual acuity in eyes with MGDA is generally poor. In the current study, 95/113 (84%) of the patients had visual acuity worse than 2/200 (counting fingers). Increased cavitary depth, relative size of excavation (the ratios of cavitary depth to axial length and cavitary depth to maximal cavitary width), and the presence of RD were significantly associated with an increased risk of poor vision. Cavitary width, product of cavitary depth and width, and the presence of PFV were not significantly associated with the risk of poor vision. Cranial abnormalities were not significantly associated with the clinical and echographic characteristics of MGDA (data not shown). One explanation is that this may be due to the limited number of patients. A larger scale of samples to define the associations better will be needed.

RD is one of the most common complications in eyes with MGDA and occurred in 129 (48%) of 271 eyes in this series. This is higher than a previous study, which reported 37% RD in eyes with MGDA (9). The higher percentage of RD observed in the current study could be partially due to the fact that this cohort of patients was collected in a tertiary referral center, which tended to treat patients with more severe and complicated conditions.

The surgical management of RD in eyes with MGDA is challenging. Rhegmatogenous RD is easier repaired than exudative RD, and the latter is easier to progress into total RD and re-detach after surgery (11, 15–17). In the current series, only three patients were diagnosed as rhegmatogenous RD, and the rest of RD eyes were exudative. Given the poor prognosis after surgery, only a small number of eyes underwent surgical intervention, and the remaining eyes were suggested regular follow-ups.

Thus, identifying relative risk factors of RD in MGDA eyes is needed for surgeons, particularly in the preverbal or minimally communicative children. The cavity produced at angential dragging force of retina around the disc and increased the risk of retinal brittleness. It is conceivable that the size and the shape of the cavity contribute to the risk of RD. In this study, we used the cavitary depth to axial length and cavitary depth to cavitary width ratios to represent the shape and the size of the cavity and found that the cavitary depth to the axial length ratio of more than or equal to 0.25 was associated with an increased risk of RD. This suggests that relative size of excavation in MGDA may prognosticate the risk of RD.

The pathogenesis of RD in MGDA has not been fully known. There are few reports about the components of RD in MGDA in large series. In previous reports, retinal breaks in MGDA may occur in the excavation and are difficult to be found (15, 18, 19). The traction of vitreous and preretinal glial tissues has been reported to be associated with the retinal breaks (18, 20). In this study, we hypothesized that deeper excavation with a sharper turn angle around the edge of the excavation would be subject to increased vitreoretinal traction and increased retinal breaks. Although the angle of edge along the excavation was not measured, size parameters of the excavation were measured in this study. The finding that cavitary depth to the axial length ratio of more than or equal to 0.25 was associated with an increased risk of RD *via* ultrasound supports this hypothesis. A prospective-design and higher-powered study would be warranted to confirm this finding.

It is worthwhile to mention that the presence of PFV was not significantly associated with poor vision and RD. In Venincasa's study, eyes with structural abnormalities may have worse vision or increased risk of RD (12). Although MGDA eyes with PFV have a higher incidence of complications (8), there was only an increasing trend toward the association between the presence of PFV and poor vision. Although we counted the numbers of the anterior, combined, and posterior types of PFV separately, the severity of proliferation was not evaluated. Thus, a prospective and higher-powered study about the association between the presence of PFV and RD would be required.

Some limitations of this study are that it was retrospective. Given the variable morphology of the excavation, volume of excavation was not calculated *via* a formula. The product of cavitary depth and width could not be equal to the volume. A multimodal approach such as three-dimensional reconstruction would be better to evaluate the morphology of the excavation. Besides, the examiners to produce the images were different in our ophthalmic center over a long period. Although the same examiner measured each excavation and axial length, some patients only had printed images available, while others had digital images. Furthermore, given the young population, assessments of visual acuity were acquired in just 42% of the eyes included.

To the best of our knowledge, this is the first report to analyze echographic measurements of MGDA eyes in a large cohort. We also correlated the echo parameters with the risk of poor vision and RD and found a more sensitive factor. MGDA with cavitary depth to the axial length ratio of more than or equal to 0.25 may have a higher risk of RD. Further clinical research will be required to confirm the effectiveness of echographic measurements to predict RD risk.

## Data Availability Statement

The original contributions presented in the study are included in the article/supplementary material, further inquiries can be directed to the corresponding author/s.

## Ethics Statement

The studies involving human participants were reviewed and approved by the Institutional Review Board of the Xinhua Hospital Affiliated to Shanghai Jiao Tong University School of Medicine. Written informed consent to participate in this study was provided by the participants' legal guardian/next of kin.

## Author Contributions

All authors contributed to the production of this manuscript and have approved the final version.

## Funding

This study was supported by the Shanghai Sailing Program (No. 20YF1429700) and the Clinical Research Plan of SHDC (No. SHDC2020CR5014-002).

## Conflict of Interest

The authors declare that the research was conducted in the absence of any commercial or financial relationships that could be construed as a potential conflict of interest.

## Publisher's Note

All claims expressed in this article are solely those of the authors and do not necessarily represent those of their affiliated organizations, or those of the publisher, the editors and the reviewers. Any product that may be evaluated in this article, or claim that may be made by its manufacturer, is not guaranteed or endorsed by the publisher.
